# P-1491. Practice variations and clinical outcomes of Intravenous Aminoglycosides in Nontuberculous Mycobacterium infections at three tertiary hospitals in London, Ontario, Canada

**DOI:** 10.1093/ofid/ofae631.1661

**Published:** 2025-01-29

**Authors:** Esraa Babaeer, Jeffrey Minuk, David McCormack, Marco Mura, Inderdeep Dhaliwal, Sameer Elsayed, Lise Bondy, Megan K Devlin, Sarah Shalhoub, Mahshid Mohammadi, Huma Saeed, Michael Silverman, Jeffrey Fuller, Fatimah Almutawa, Reza Rahimi Shahmirzadi

**Affiliations:** Western University, London Ontario, London, Ontario, Canada; Western University, London, Ontario, Canada; Western University, London Ontario, London, Ontario, Canada; Western University, London Ontario, London, Ontario, Canada; Western University, London Ontario, London, Ontario, Canada; Western University, London, Ontario, Canada; Western University, London, Ontario, Canada; Western University, London, Ontario, Canada; LHSC/Western University, London, Ontario, Canada; Western University, London Ontario, London, Ontario, Canada; University of Western Ontario, London, Ontario, Canada; Western University, Lawson Health Research Institute., London, Ontario, Canada; Western University, London Ontario, London, Ontario, Canada; Western University, London, Ontario, Canada; Western University, London, Ontario, Canada

## Abstract

**Background:**

The worldwide occurrence of nontuberculous Mycobacterium (NTM) infections is rising. In Canada, there is a continuing surge in infections caused by Mycobacterium avium complex (MAC). Treating NTM infections is challenging and requires multidrug regimens with antimicrobial susceptibilities to guide therapy.

Generally, aminoglycosides have moderate in-vitro activity against NTM isolates. However, the synergistic effect with other antimicrobials makes it an attractive choice for clinicians, especially in the management of severe or refractory cases. The recommended duration of intravenous aminoglycosides is debatable due to side effects and the need for therapeutic monitoring. Moreover, NTMs are becoming more resistant to aminoglycosides.

This study aims to assess clinical practice variations in aminoglycoside use among NTM infections in terms of duration and monitoring in London, Canada.

Patient Characteristics and Clinical Presentation

**Figure:**
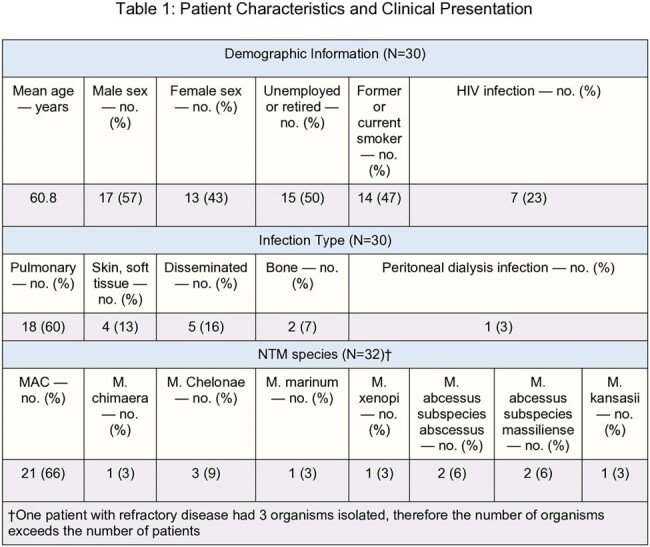

**Methods:**

Retrospective study reviewing the characteristics of 30 patients diagnosed with NTM infection between January 1, 2019 and December 31, 2023 in three tertiary care hospitals in London, Ontario, Canada.

**Table 2:**
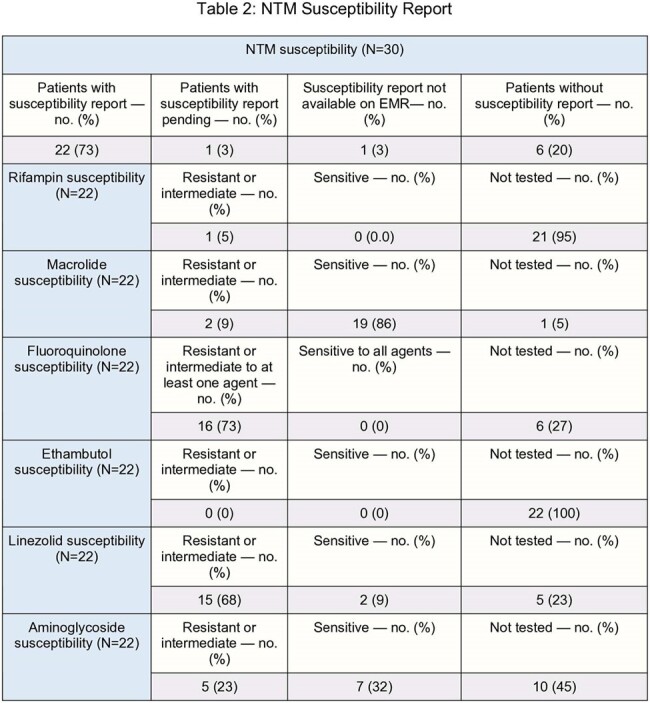
NTM Susceptibility Report

**Results:**

Mean age at diagnosis was 60.8 years. Seventeen (57%) were male and 13 (43%) were female. The presentations included pulmonary NTM infections in 19 (63%) and skin and soft tissue infections in four (13%). Among all identified species, MAC was the most common.

Fifteen (50%) patients received IV aminoglycosides, with 7 (32%) having documented susceptibility to aminoglycosides. Duration of aminoglycoside use varied among patients, ranging from 2 to 24 weeks. Of the patients started on an IV aminoglycoside, 3 (20%) developed ototoxicity and 2 (13%) had acute kidney injury. Serum aminoglycoside levels were checked in 12 (80%), but only 7 patients (47%) had at least one audiology screen.

In terms of outcomes, 7 (23%) achieved clinical cure while 9 (30%) had refractory disease, 7 (78%) of which were pulmonary. Two (57%) of the cured patients and 2 (22%) of the refractory patients had received an aminoglycoside.

Aminoglycoside Use and Monitoring

**Figure:**
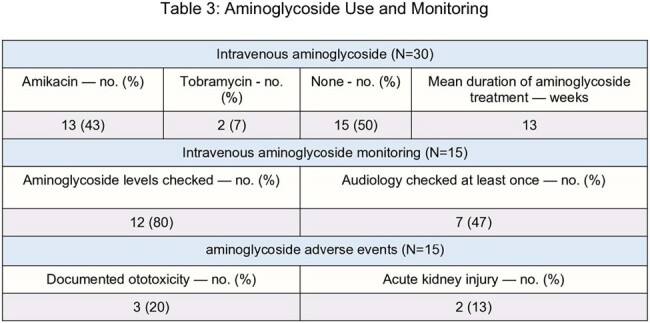

**Conclusion:**

Aminoglycoside use for the treatment of NTMs is variable among clinicians, mostly due to concerns for side effects and the need for consistent drug monitoring, which limits its implementation in outpatient settings.

Clinical Outcomes

**Figure:**
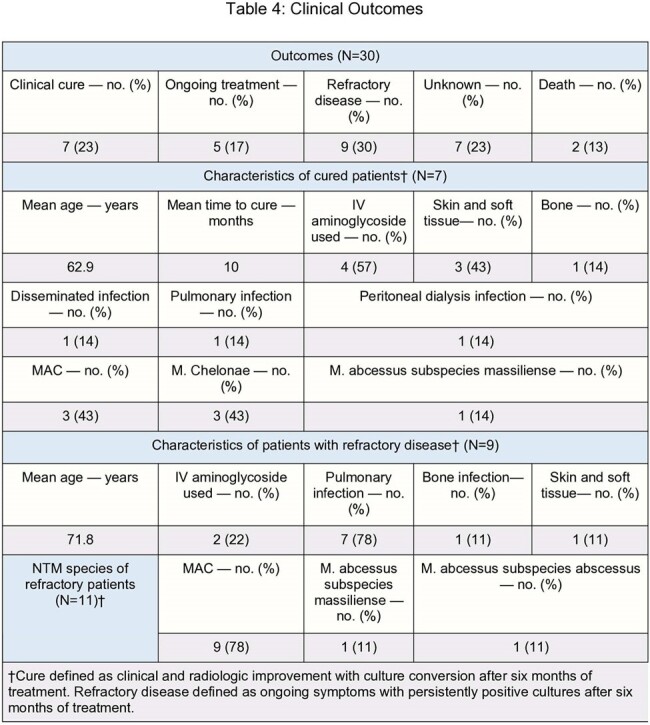

**Disclosures:**

**Lise Bondy, Assistant Professor**, Pfizer: Honoraria|Viiv: Advisor/Consultant **Michael Silverman, MD, FRCP, FACP, AAHIVMed**, Pfizer: Grant/Research Support

